# Book Notice

**Published:** 1923-10

**Authors:** 


### BOOK NOTICE

The following is taken from a very interesting book just received from Dr. A. Bouland, of Paris. All the book is in French but the part given below. This article relates to practice of Dentistry in France.

### STUDIES RELATING TO THE DIPLOMA OF SURGEON-DENTIST, AND CONDITIONS FOR EXERCISING DENTISTRY IN FRANCE

### Special regulations in regard to foreigners.

Special regulations relating to foreigners were already provided by article 7 of the Law of November 30, 1892. These regulations have been modified by article 19 of the decree of January 10, 1909, reading as under:

Dentists qualified abroad, who desire to exercise their profession in France are obliged to pass the examinations provided by the present decree.

From November 1, 1909, they may obtain exemption from the obligatory course of study, and partial exemption of scholarity, after notification of the consultative Committee of Public Instruction, provided they possess the baccalaureate of secondary education, or a certificate of matriculation, or a certificate of primary superior studies.

In regard to foreigners, we have to consider those who enter France to carry on their studies with a view of establishing themselves in France on the completion of their studies; those who, having a diploma in their own country, come to France in order to exercise their profession, and finally those who with or without a diploma in their own country come to France, in order to pursue their studies with the view of obtaining a University degree, having no legal value, from which, however, they may draw advantage in the country of their origin, or with the simple object of following their studies without seeking diplomas.

In the sequence, it will be observed how different the rules of study are for these three categories of foreign students.

### FOREIGN STUDENTS DESIRING TO ESTABLISH THEMSELVES IN FRANCE OF THE COMPLETION OF THEIR STUDIES

Those students who desire to settle in the capital or the French Colonies, when their studies are finished, are subject, of course, to the same studies, as the French students, since the state diploma, which crowns their studies, confers on them the same rights.

The regulations provided by the decrees of January 11, 1909, and subsequent decrees are, therefore, applicable to those students stence, they have to accomplish five years of studies (two years obligatory course, and three years scholarity). If these students are ex-soldiers of allied countries, or friends of France, they may benefit by special measures.

They must, in addition, to enable them to be admitted to the obligatory course of study (le stage) to have reached the age of sixteen years, and according to the decree of January 11, 1909, must prove possession of the baccalaureate, certificate of matriculation, or certificate of superior primary instruction, or for young ladies, one of the three qualifications named above, or the certificate for the final studies in secondary education.

### FOREIGN DOCTORS AND SURGEON-DENTISTS DESIRING TO EXERCISE THEIR PROFESSION IN FRANCE

The exercise of dentistry in France is regulated by the Law of November 30, 1892; the decree of January 11, 1909, and subsequent ones lay down the conditions of study for the diploma of Surgeon-Dentist.

The diplomas, patents, licenses conferring the right of legally exercising the profession of dentistry in different countries possess no value whatever in France, and do not afford any right to carry on dentistry upon the territory of the French Republic, or her Colonies.

The only diplomas conferring the right of legally exercising the dental profession, are:

The French state diploma of Doctor of Medicine;

The French state diploma of Surgeon-Dentist;

The diploma of the Alsations and Lorrainers having acquired validity for the exercise of dentistry, upon the entire French territory, by the Law of July 13, 1921.

In passing, it may be again mentioned, that the right of exercising dentistry is maintained for those put on the dental register in France by patent, on January 1, 1892.

Foreign doctors or dentists, whatever may be their acquirements or degrees, can not, therefore, carry on their profession in France, except they have obtained the French diploma of doctor of medicine, of Surgeon-Dentist.

We have seen that the studies for obtaining the diploma of Surgeon-Dentist cover a minimum period of five years. Foreigners wishing to practice dentistry in France, are they to continue their studies for five years, like the young students?

Article 19 has provided for eventual reductions in the period of the obligatory course and of scholarity.

At the outset one essential point must be borne in mind, that if, as will be seen further on, a reduction is made for the candidate in the duration of the studies, it can have no reference to equivalents, nor to exemption from examinations.

The Foreign dentist must first of all acquire one of the French qualifications required of regular students: the baccaleureate, a certificate of matriculation, or the certificate of superior primary education.

A good knowledge of French will be indispensable for the candidate as the examinations are held in French.

One of the qualifications cited above having been obtained, the candidates may obtain exemption of the obligatory course of study (stage, two years) and partial relief of scholarity (one to two years), after notification of the consultative committee of Public Instruction of France, which may reduce the studies for these candidates to three, two or one years. The candidates, even should the obtain the reduction of scholarity, are compelled to undergo all the examinations provided for the students carrying out the regular studies: Examination as to compulsary course (stage) four tests.

1° Examination of scholarity (1 oral test).
2° — (1 oral test).
3° — (3 practical tests).

The candidates desirous of profiting by the reductions of studies provided in favor of foreigners possessing diplomas, must address their request to the Minister of Public Instruction in France.

This request must, of course, be accompanied by documentary proofs, such as extracts from certificates as to educational qualifications, copies of diplomas, etc.

The Minister of Public Instruction, and the consultative Committee of Public Instruction will decide upon each candidates case after examination of the proofs bearing upon it, viz.: studies, university diplomas, work, etc. The candidates, having served in the allied armies, may usefully add particulars of their military service.

STUDENTS ENTERING FACULTIES OR SCHOOLS, IN ORDER TO COMPLETE THERE THEIR UNIVERSITY STUDIES

Young persons coming from abroad to our Faculties or Schools in order to pursue their studies without any intention of fixing themselves in France, will benefit by a special regulation.

These students come to us for the purpose of gaining professional instruction which will be of service to them in their own country.

They may obtain divers diplomas, as for instance:

Diploma of Surgeon-Dentist of the University.

The Faculties of Nancy, Lille and of Bordeaux issue diplomas of Surgeon-Dentist of the University. These diplomas do not accord the right of exercising dentistry in France.

### FACULTY OF MEDICINE OF NANCY AND LILLE

Students who are foreigners, in registering for the acquirement of the diploma of the University, must produce the degrees they hold from the University of their countries. They are allowed to register after notification of the faculty, that the degrees submitted have been judged to be equivalent by the consultative committee of Public Instruction and with the concurrence of the Minister of Public Instruction.

The studies and the examinations are the same as for the state diplomas, the fees for the studies are the same, except as to the rights of the certificates of aptitude and of the diploma.

The diploma at the conclusion of the studies will be that of Surgeon-Dentist of the University.

### FACULTY OF MEDICINE OF BORDEAUX

The Faculty of Bordeaux, likewise issues a diploma of Surgeon-Dentist of the University to Foreigners. The candidates for this diploma:

1° If they have pursued anterior studies, or studies carried out wa Foreign Faculty, must forward a request for admission to the Dean of the Faculty of Medicine of Bordeaux, indicating the nature of their foreign diplomas.

2° If they have never followed any studies in default of a University diploma, they must bring certificates that they have practiced dentistry or certificates that they are dental mechanicians.

All documents are examined and the result, accordingly, will be rejection of the request, or admission is granted and the period for study indicated.

### DIPLOMAS OF THE INDEPENDENT DENTAL SCHOOLS

Certain foreign students also come to the Dental Schools for professional instruction and a diploma, which from the legal standpoint has no value whatever in France, but may confer on them advantages, perhaps even the right to practice in certain countries. These students come to carry on their studies, other to complete studies, which they have already commenced.

The conditions for admission, the duration of the studies, the fees for the studies, are variable in regard to the schools and are set out in their respective calendars.

